# Effects of Different Loading Programs on Finger Strength in Rock Climbers

**DOI:** 10.1186/s40798-024-00793-7

**Published:** 2024-11-19

**Authors:** Natalie K. Gilmore, Peter Klimek, Emil Abrahamsson, Keith Baar

**Affiliations:** 1 Department of Neurobiology, Physiology and Behavior, University of California Davis, Davis, CA 95616 USA; 2 Crimpd, Inc., Seattle, WA 98118 USA; 3 Stones & Stories, Årsta, AB 120 52 Sweden; 4 Department of Physiology and Membrane Biology, University of California Davis, One Shields Ave, 195 Briggs Hall, Davis, CA 95616 USA; 5 VA Northern California Health Care System, Mather, CA 95655 USA

**Keywords:** Rock climbing, Exercise, Training, Force transfer, Neurological strength

## Abstract

**Background:**

Climbing places high loads through the hands and fingers, and climbers may benefit from specific finger strength training (hangboarding) protocols. The purpose of this study was to evaluate the effect of a 10-minute low intensity hangboard finger strengthening protocol (“Abrahangs”), compared with the generally accepted Max Hangs protocol for training maximal grip strength.

**Methods:**

We retrospectively evaluated the change in grip strength and Strength: Weight following Max Hangs, Abrahangs, or the two protocols performed concurrently in rock climbers who used the Crimpd app to log their training. Users who had completed two finger strength tests within a 4–16-week period were included. Climbers were grouped by the number of training sessions into: “Climbing Only”, “Abrahangs Only”, “Max Hangs Only” and “Both” Max Hangs and Abrahangs.

**Results:**

Frequent low intensity finger loading was as effective at improving grip strength in climbers as training with maximal loads. Additionally, combining low intensity and maximal load training resulted in additive strength gains.

**Conclusions:**

These results suggest that low-intensity long duration holds provide a promising training paradigm for training finger strength that is gentle enough to incorporate into existing training programs.

## Key Points

**•** A 10-minute low-intensity long duration hold protocol can improve finger strength in rock climbers.
**•** The increase in strength with low-intensity long duration holds is equivalent to that of maximal hang training.
**•** The two types of training have an additive effect on grip strength.
**•** This is the first retrospective study to examine the effects of different training programs in a large population of climbers.

## Background

Rock climbing has become increasingly popular worldwide. It was one of the fastest-growing sports even before its introduction into the Tokyo 2020 Olympics. Climbing evolved from mountaineering with the ascent of natural rock faces, and the sport has since evolved into several unique disciplines. Today, these disciplines include outdoor climbing, which is split into the main disciplines of bouldering, sport climbing, and traditional (trad) climbing; and indoor climbing using artificial holds and training in gyms, with disciplines including bouldering, lead, top rope, and speed climbing. The man-made indoor climbing routes provide a more controlled environment that is helpful for standardized training and competition. For example, many climbing gyms contain common training equipment such as hangboards, campus boards, and universal training boards (i.e. MoonBoard, Tension Board, Kilter Board) that allow for a higher level of standardization than is possible through climbing alone, whether that be outside or indoors.

A rise in intensity and training has accompanied the growth of the sport and has led to an increase in the number of goal-oriented climbers using supplemental training to improve strength and boost performance. It is important to scientifically evaluate the efficacy of training protocols to ensure climbers can continue to push the boundaries of the sport by implementing effective training methods.

Climbing engages the whole body, but the unique demands of the sport place particularly high stress on the hands and fingers [1]. The greatest predictors of climbing ability are maximal strength and endurance of the finger flexor system [2]. As there is not significant muscle tissue within the fingers themselves, the key tissues involved in finger strength include the muscles of the forearm, flexor tendons, finger annular pulleys, volar plates, and ligaments [3]. Bones, tendons, and ligaments have generally been thought to adapt much more slowly to training than muscle. It is commonly accepted that finger strength in climbers takes years to build, and injury occurs when the forces placed on these tissues exceed their biomechanical force tolerance [34]. Contrary to this belief, Smeets and colleagues found that tendon, ligament, and bone protein turnover rates are similar to, or greater than, those of muscle [5]. In their small study (*n* = 6) of older patients undergoing total knee replacement, the rate of protein turnover was greater in the anterior cruciate ligament and patellar tendon than in the quadriceps muscle. Additionally, in-vitro loading of engineered ligaments also indicates that hormonal signals produced by exercise [6] and mechanical signals resulting from loading [7] rapidly stimulate collagen production and increase ligament tensile strength. However, this model is developmentally immature with far more cells and far less collagen than human tendons [8]. So, whether a similar response would be seen in humans remains an open question. The idea that the strength of the flexor system connective tissues – tendon, ligament, and fascia – could adapt more quickly than previously appreciated if the proper stimulus is delivered and sufficient rest provided is attractive to climbers.

Connective tissues of the musculoskeletal system provide mechanical support and play key roles in moving the skeleton [9]. Tendons, ligaments, and fascia are dense connective tissues: tendons link muscle to bone and transmit muscle force to the skeleton, ligaments link bone to bone to stabilize joints, and fascia organizes tissues and promotes movement [10]. The connective tissues begin as a single cell type that adapt postnatally to the load placed on them [11]. This means that these tissues sense and adapt to mechanical forces. The primary forces within the musculoskeletal system are tension, compression, and shear [12]. In climbing, the finger flexor tendons largely experience tension. Given sufficient volume and intensity, tensional loads can increase the size and mechanical strength of tendons. This has been clearly demonstrated in the patellar tendon of fencers and badminton athletes [13], as well as the finger flexor system in climbers [14]. Importantly, tension is not the only force experienced by the finger flexor system. Both compression and shear forces are present in the regions of the flexor tendons that slide under the flexor pulley system (palmar aponeurosis, and annular and cruciform pulleys).

Climbing leads to adaptations of connective tissue within the hands and fingers. Specific adaptations that have been previously noted include hypertrophy of finger flexor tendons and ligaments, annular pulleys, and volar plates, which have been shown to occur in climbers within a time scale of several years [1, 3, 15, 16]. Previous literature notes significant hypertrophic adaptations in connective tissue of the hands and fingers of climbers with greater than 15 years of experience. The greatest increases in thickness occur in the A2 and A4 pulleys (a 63% and 68% respective increase in thickness on average for all digits compared to non-climbers) which take on the greatest biomechanical loads of the annular pulleys in the crimp position [17, 18]. The joint capsule of the distal interphalangeal joint also is ~ 40% thicker in experienced climbers [16]. Lastly, the flexor tendons of climbers have been measured to be up to 18% thicker than those of non-climbers [4]. These data suggest that rock climbers may be a unique population for understanding the mechanism of tendon adaptation to training. Climbing places unique demands on the finger tendons and pulleys. The Achilles and patellar tendons have adapted to carry the weight of the body by growing in cross-section to minimize the stress on the tissue. By contrast, climbing is one of the few circumstances in which the weight of the body passes through the small tendons of the hand, resulting in much higher stresses. The result is both adaptations [16] and overuse injuries not seen in other sports.

With the increase in interest and intensity, the prevalence of injuries to flexor tendons and finger pulleys has also escalated. A retrospective cross-sectional study from 2007 found that approximately half of climbers had experienced a climbing-related injury within the past year: 10% of those injuries resulted from falls, 33% from chronic overuse, and 28% due to acute injury from performing a strenuous movement [19]. Fingers are the most common location of overuse and acute overstrain injuries in climbers [19], with approximately 60% of all climbing injuries involving the hand and fingers [17]. The sites within the fingers and hand most prone to injury in climbers are the annular pulleys, flexor tendons, finger collateral ligaments, and bones [1, 20]. Volar plate injuries are also common in adolescent climbers [1]. Treatment of tendon and ligament injuries is limited and often has unfavorable long-term functional outcomes [21]. Therefore, it is vital to develop physiologically sound climbing training methods to boost connective tissue adaptations while minimizing injury risk and protecting from damage.

The mechanical stress from load placed on tendons and ligaments during exercise activates collagen synthesis and causes connective tissue hypertrophy [13, 22, 23]. Turnover of collagen in response to training in adult tendon occurs on the outer surface, indicating that training drives deposition of new collagen on the outer layers of the tendon [24]. Our group used engineered human ligaments to show that the adaptive molecular response is independent of the intensity of loading (even a light load resulted in enhanced collagen synthesis) and that repeated short bouts of loading (10 min of activity every 6 h) provided the optimal stimulus for enhancing connective tissue strength [22].

Climbers commonly train finger strength using hangboarding, also known as fingerboarding. Hangboards are used to load the finger pulley system in a controlled manner. Most protocols are based on one of two goals: improving grip strength or minimizing edge size [25]. Hangboarding significantly improves maximal finger strength, explosive strength, and endurance faster than climbing on its own [1, 2, 10, 26, 27]. While climbing alone can lead to long-term finger strength and tissue adaptations, studies of shorter duration consistently show that hangboarding produces positive effects in interventions as short as 4–10 weeks (Levernier & Laffaye [27] and Hermans et al. [2], respectively). These short hangboard training studies compare an intervention to a climbing-only group, which consistently show no improvements in finger strength, to demonstrate that targeted short-term interventions can improve performance. The findings suggest that targeted training can lead to more rapid finger strength gains than climbing alone. Hangboarding can be performed with less than body weight, body weight, or more than body weight allowing the athlete fine control over the load [3]. Even though many techniques can improve grip strength, Max Hangs, performed between 80 and 95% of the athlete’s one-rep maximum (1RM), are the most widely used program for climbers to increase maximum force production and improve climbing performance [2, 25]. This practice is consistent with the idea that heavy load is needed to increase maximal isometric grip strength [28, 29, 26]. While Max Hangs are very effective for training maximal force production, the high intensity may carry risks of finger overload injury. This is especially a problem for novice climbers who attempt high intensity loading with limited loading history [3].

Many different protocols have been used in scientific studies to assess finger strength in climbers; however, these protocols vary broadly depending on which component of strength they measure (i.e. strength, endurance, or power) and there are no established standard testing procedures [30, 31]. Most climbers measure finger strength using an intermittent loading to failure test with edge depths between 20 and 30 mm, work times between 5 and 10 s and rest periods between 2 and 5 min [31]. By incrementally adding load until failure, this test allows for tracking strength-to-weight ratio in climbers as a function of time in each individual.

Isometric loading has previously been shown to have positive effects on tendon in the management and treatment of tendinopathy. Using a surgical model of patellar tendinopathy in the rat, we have previously demonstrated that a single bout of isometric exercise caused an increase in the expression of genes that regulate tendon regeneration including scleraxis and collagen Ia1 whereas a time-under-tension match dynamic load resulted in the expression of cartilage genes such as collagen IIa1 [32]. In athletes with patellar tendinopathy, daily isometric loading for four weeks before progressively returning to isotonic, explosive, and finally sport-specific exercises was associated with better pain reduction and return to sport compared with an eccentric loading control group [33].

Taking inspiration from the research published by our group on the adaptive capacity of connective tissue [7, 23], professional climber Emil Abrahamsson and his brother Felix designed a 10-minute sub-maximal hangboard program. Emil discussed the protocol (which we will refer to in the present study as “Abrahangs”) on a YouTube post (https://shorturl.at/OWXY8) that has more than a million views. With the popularity of the program, the climbing app Crimpd added “Emil’s Sub-max Daily Fingerboard Routine” to their training protocols. For the Abrahangs training protocol, all hangs are performed at a low intensity (with feet on the ground, the climber loads until they feel a “light strain on their forearms, ~ 40% of max”), and users are advised to perform the exercises with at least a 6-hour window between workouts or climbing sessions, in conjunction with the refractory period for maximal activation of tendon adaptation biochemical pathways. However, the ability of this protocol to increase grip strength and physiological adaptations has yet to be tested scientifically.

The goal of the current study was therefore to retrospectively evaluate the strength training benefits of the Abrahangs protocol in relation to a broadly used Max Hang protocol. To begin to address this goal, we queried existing data from climbers who used the Crimpd climbing app to test their finger strength between September 2022 and December 2023. Participants were grouped by those who only climbed and those who performed Max Hang training, the Abrahangs protocol, or combined Max Hangs with Abrahangs. Users were not guided toward completing any specific protocol and we do not know the motivation for a user to choose a particular protocol, nor did we control for other potential confounding variables or activities performed by participants during the training window.

## Methods

Our retrospective training data were obtained by compiling user-logged workouts in the Crimpd app, and then filtering the data to analyze the users that met our selection criteria. Participant demographics are listed in Table 1. Each assessment and training bout logged was completed without a determined or suggested schedule. We analyzed data from only the individuals who voluntarily completed two assessments in a 4–16-week period and also fit our other criteria (including performing above the minimum cutoff of Abrahangs or Max Hangs workouts between assessments). This means that many more app users have completed the workouts, but without their self-reported assessment data, we could not assess their strength changes and thus did not include these individuals. Table 2 contains data on the length of the testing window as a function of the percent of climbers in each group, split into 4-week increments. Our retrospective data set does not include information on prior training history, so it is unknown whether the climbers in this study were new to or continuing with these hangboard training protocols. Table 3 summarizes the general parameters of each training protocol. For each protocol, climbers were given specific prompts in the Crimpd app for how much effort and strain they should aim to achieve. Additionally, the app interface includes instructional videos educating participants on proper form/grip position for each exercise. Participants used the in-app timer to work through the protocols. While this does not guarantee that participants performed the workouts/assessments exactly as intended, we believe this provides real world applicability for the protocol since all participants had equal access to these instructions.

### Protocol Links

Abrahangs: https://tinyurl.com/2wxbpyd2.

Max Hangs: https://tinyurl.com/a225xh7d.

Finger Strength Assessment: https://tinyurl.com/2d5d9dne.

**Table 1:**
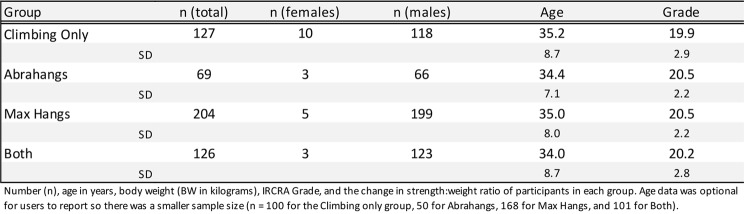
Participant demographics. The “Both” group performed both Abrahangs and Max Hangs exercises

**Table 2:**
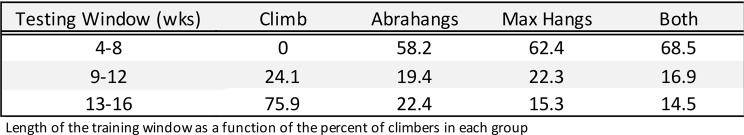
Length of testing window by group. The “Both” group performed both Abrahangs and Max Hangs exercises

**Table 3:**
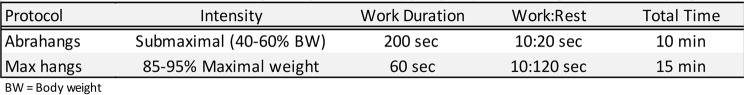
Parameters of each training bout performed by per group

### Finger Strength Test (1RM) Protocol

**Fig. 1:**
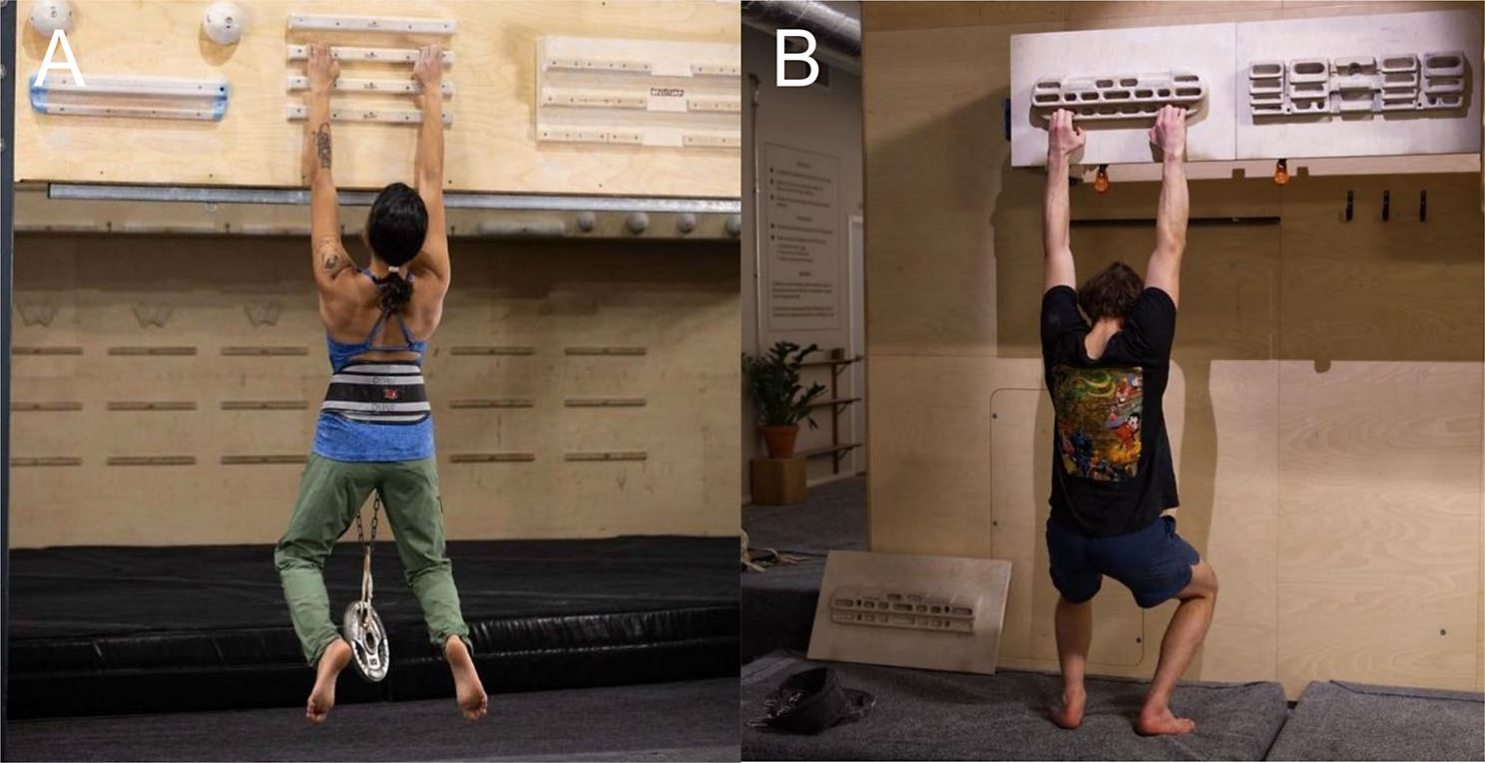
Demonstration Abrahangs and Max Hangs. These exercises can be performed on an 18–22 mm edge of a hangboard. **A**) A dead hang with added weight representative of the Max Hangs and Finger Strength Test (1RM) protocol and **B**) An isometric hang performed with feet on the ground representative of the Abrahangs protocol

The finger strength test was performed on the user’s own hangboard using a ~ 20 mm edge with the hand in a strict half crimp position (Fig. 1A), with arms straight or bent slightly. The climber completed up to 8 sets of hangs with progressively heavier loads until failure. Participants were instructed that each weight must be held for 7 s to count. Two minutes of rest were permitted between sets. If the climber could not hold their body weight, weight was removed from the body using a pulley system and counterweight, and the counterweight was subtracted from body weight. To add weight above body weight, the climbers were instructed to attach weights to their body using a climbing harness (Fig. 1A). Load was increased slowly, with a maximum of 5 kg added for each increment. If failure load was not reached within 8 sets, the climber completed the test on another day from a higher starting load.

### Training and Maximum Strength Testing Protocols

#### Max Hangs Protocol

Max Hangs training was performed on a 20 mm deep edge, like the finger strength test (Fig. 1A). The load for this protocol could be defined by the climber as 85, 90, or 95% of the total weight obtained from the Finger Strength Test. For this study, the data from each of these three separate protocols were pooled. The training protocol consisted of dead hangs with 85–95% of 1RM for six 10-second repetitions, with a rest of two minutes between repetitions.

#### Abrahangs Protocol

Abrahangs were performed using an edge 18–22 mm deep and the climbers were instructed to keep their feet on the floor at all times (Fig. 1B). Climbers were instructed to keep the intensity low by providing the cue that “only a small strain should be felt in the forearms during the hang”. The specific hangs for the protocol were:

Isometric Hang: (1 set x 6 reps x 00:10 per rep), rest 00:20 per rep.

Isometric Hang: Front 3 (using digits 2–4) Open (1 set x 6 reps x 00:10 per rep), rest 00:20 per rep.

Isometric Hang: Front 2 (digits 2 and 3) Open (1 set x 2 reps x 00:10 per rep), rest 00:20 per rep.

Isometric Hang: Middle 2 (digits 3 and 4) Open (1 set x 2 reps x 00:10 per rep), rest 00:20 per rep.

Isometric Hang: Front 2 Half Crimp (1 set x 2 reps x 00:10 per rep), rest 00:20 per rep.

Isometric Hang: Middle 2 Half Crimp (1 set x 2 reps x 00:10 per rep), rest 00:20 per rep.

### Selection Criteria and Participant Demographics

Both males and females were considered in this study, but the total number of female participants (*n* = 21) was far fewer than the number of males (*n* = 506), because the population of climbers who regularly train and log finger strengthening workouts on the app skews heavily male. The male and female data were analyzed together since the female group would lack sufficient power to draw conclusions (Table 1). Age was optional to report and Table 1 contains age for users who opted to self-report (*n* = 100 for the Climbing only group, 50 for Abrahangs, 168 for Max Hangs, and 101 for Both). Δ Strength: weight ratio difference (%) was reported (Table 4; Fig. 3) for each intervention group. Table 4 contains the mean resistance and body weight for each group during the pre- and post-test finger strength assessments. A Robust progession and OUTlier Removal (ROUT) outlier test was performed on the Δ Strength: Weight ratio difference data that led to the exclusion of a total of 10 climbers (*n* = 1 for the climbing only group, *n* = 3 for Abrahangs, *n* = 4 for Max Hangs, and *n* = 2 for Abrahangs + Max Hangs) from our analysis. Effect size is reported for the Δ Strength: Weight ratio difference in Table 5: Cohen’s *d* was reported for each intervention compared to the Climbing Only group.

**Table 4:**
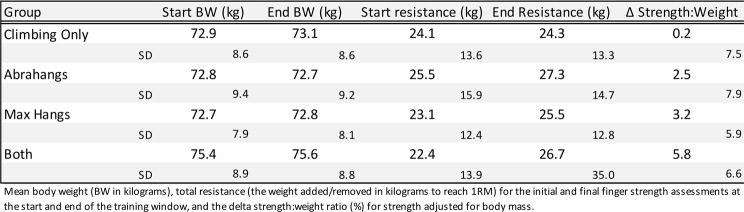
Assesment strength and body weight. The “Both” group performed both Abrahangs and Max Hangs exercises

**Table 5:**
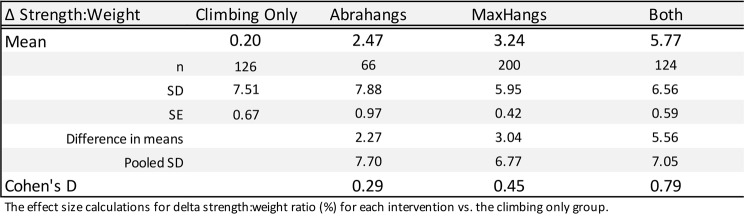
Effect size (Cohen’s *d*) of Δ Strength: Weight for each training intervention. The “Both” group performed both Abrahangs and Max Hangs exercises

**Fig. 2:**
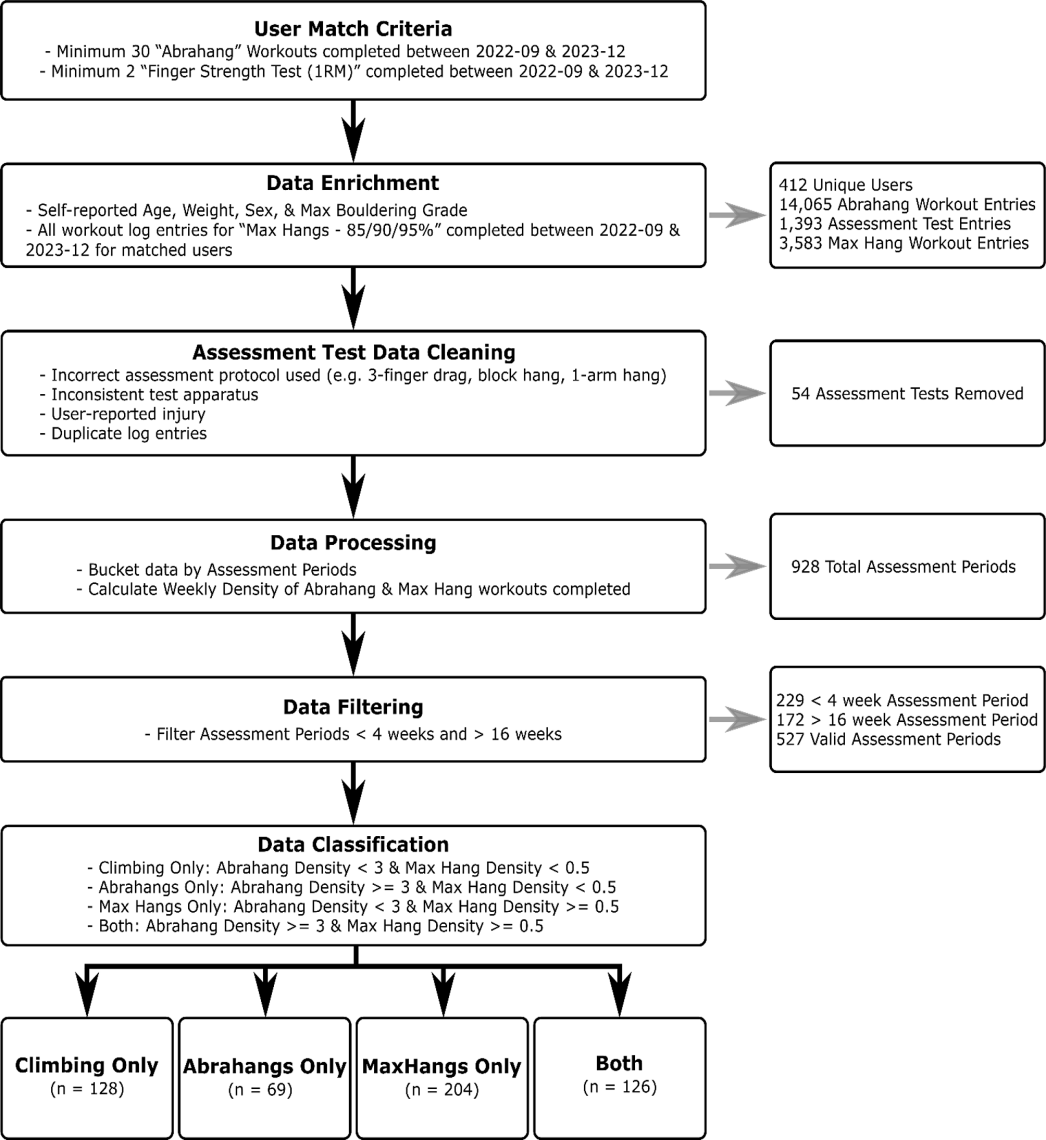
Data mining scheme used to mine users of the Crimpd app for inclusion in the study and grouping

The inclusion criteria are described in Fig. 2. Briefly, only climbers who had completed at least 30 Abrahangs workouts were included in the initial cohort. Those climbers who had also completed two Finger Strength Assessment protocols within a 4- to 16-week period were further analyzed. This 4–16-week training window was provided to increase the number of climbers who could be included. The “Climbing Only” group included users who did not complete a meaningful number (below the grouping cutoff) of finger training workouts within the assessment window. The grouping cutoffs were defined as follows: “Climbing Only” weekly average workouts during the training window of < 3 for Abrahangs and < 0.5 for Max Hangs; “Abrahangs Only” had a density of ≥ 3/wk for Abrahangs and < 0.5/wk for Max Hangs; “Max Hangs Only” had a density of < 3/wk for Abrahangs and ≥ 0.5/wk for Max Hangs; and “Both” logged densities ≥ 3/wk for Abrahangs and ≥ 0.5/wk for Max Hangs during the training window. This grouping scheme allowed for the comparison of each training method, as well as the effects of both training methods performed within the same training window. The total training, climbing volumes, and other forms of finger training were not tracked in participants.

There are several different systems used to quantify climbing performance varying across geographical location and disciplines, but the International Rock Climbing Research Association (IRCRA) recently developed the universal IRCRA Reporting Scale, which we used. Crimpd app users self-report their sex and maximum boulder grade in their preferred scale. These data were converted to the IRCRA Reporting Scale. IRCRA classification for all training groups ranged between levels 12–27. The ranges of the climbers fell between the categories of Intermediate (IRCRA level 2) to Elite (IRCRA level 4): Intermediate (*n* = 54), Advanced (*n* = 432), Elite (*n* = 38).

**Fig. 3:**
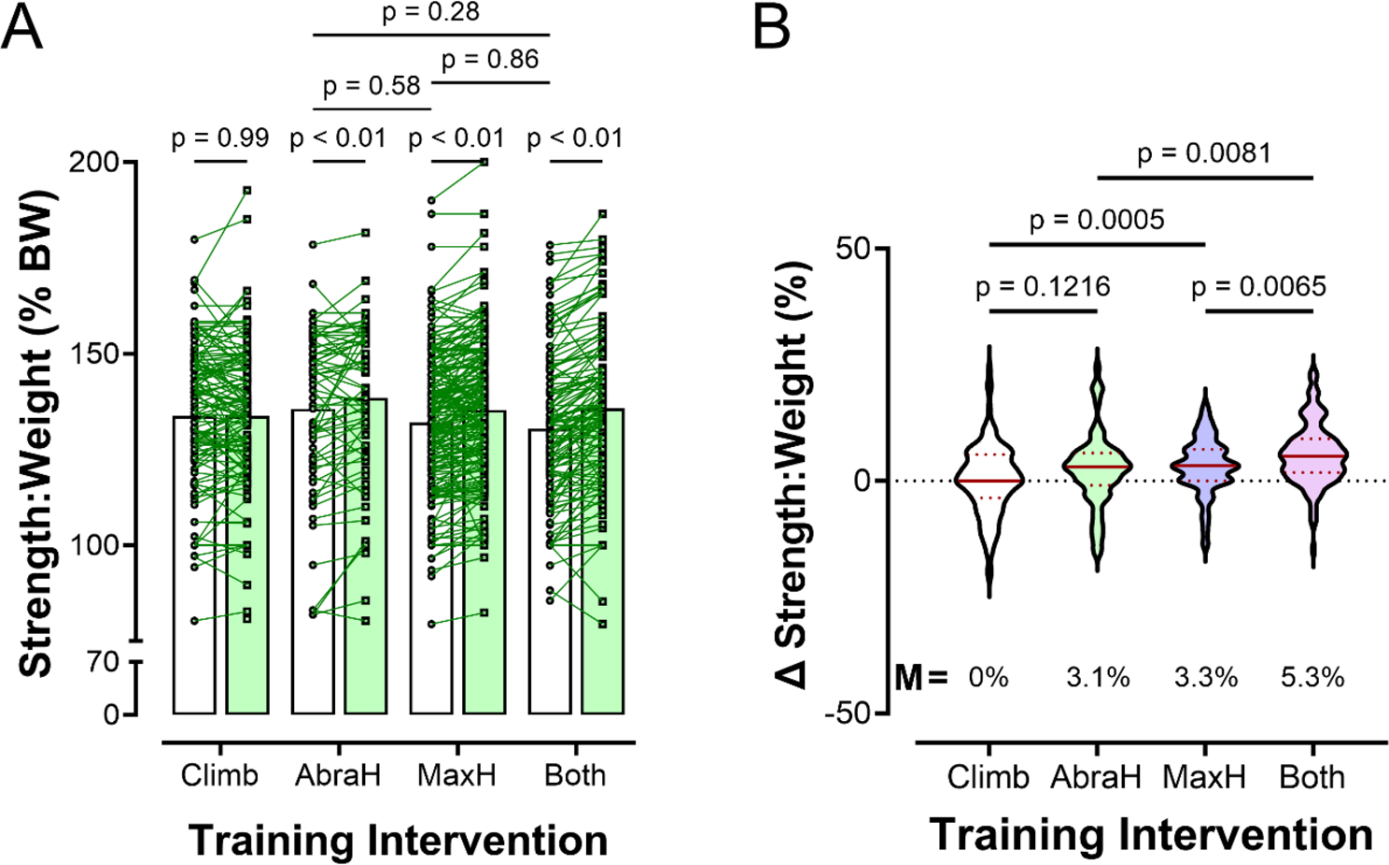
Effect of Climbing Only, Abrahangs Only (AbraH), Max Hangs Only (MaxH), and both Abrahangs and Max Hangs (“Both”) on (**A**) strength to weight ratio and (**B**) the change in strength to weight ratio. Note that all three training groups increased strength to weight ratio from the start to end of the training period, whereas those who only climbed did not increase strength. Overall, there was no difference between the training groups. However, when the change in strength was calculated, the “Both” group increased strength more than either of the groups who performed only one type of training. In panel B, the median (M) Δ Strength: Weight is reported for all groups

### Statistics

Statistical analysis was performed using GraphPad Prism, version 10.1 (San Diego, CA, USA). Group differences for the change in Strength: Weight ratio from the start of the training period to the end were compared with two-way ANOVA with time and training as the two variables. Because a significant effect was found, post hoc analysis was performed using a Tukey’s Honestly Significant Difference (HSD) test since groups demonstrated equal variance. The difference in Strength: Weight ratio was analyzed using a one-way ANOVA followed by Tukey’s HSD. Data are presented as mean and all replicates or the violin plot of all replicates are shown on the graphs. A threshold of *p* ≤ 0.05 was set a priori for statistical significance.

## Results

The maximum finger strength for all Crimpd app users across four training groups (*n* = 526) is reported in Table 4. There was no change in finger strength over the training window for the “Climbing Only” group (*p* > 0.99). A statistically significant increase in the pre-to-post strength-to-weight ratio was observed in the “Abrahangs Only”, “Max Hangs Only”, and “Both” groups (*p* < 0.01 in all groups; Fig. 3A).

We calculated the percent change in the strength-to-weight ratio (Δ Strength: Weight) between the start and end of the training window (Δ Strength: Weight (%) = (Strength: Weight Ratio End – Strength: Weight Ratio Start)/ Strength: Weight Ratio Start) x 100). The “Climbing Only” group had no change in strength within the training window. The “Abrahangs Only” group showed a positive trend with a mean increase of 2.5% and a median 3.1% increase (*p* = 0.1216; Fig. 3B). There were significant changes in strength in both the “Max Hangs Only” (mean = 3.2% and median = 3.3%; *p* = 0.0005 vs. “Climbing Only”) and “Both” (mean delta = 5.8% and median delta = 5.3%; *p* = 0.0081 vs. “Abrahangs Only”, *p* = 0.0065 vs. “Max Hangs Only”) groups. Interestingly, the improvement in the “Both” group was statistically greater than either the “Abrahangs Only” or “Max Hangs Only” groups, with the mean increase (5.8%) being the approximate sum of the mean “Abrahangs Only” (2.5%) and the “Max Hangs Only” (3.2%) groups. There was no statistical difference between the “Abrahangs Only” and “Max Hangs Only” groups. Cohen’s *d*, representing the standardized mean difference (SMD) between two groups, was reported for Δ Strength: Weight (Table 4) for “Abrahangs Only” (0.29), “Max Hangs Only” (0.45) and “Both” (0.79), with each calculated against the “Climbing Only” group.

## Discussion

This retrospective study of rock climbers showed that training frequently with low weight or training with maximum strength hangboarding significantly increased finger strength. Combining low weight and heavy training resulted in an additive effect on finger strength. Although p values can discern statistical differences between groups, these differences may not necessarily be practically meaningful. Effect size coefficients, such as Cohen’s *d*, can be used to further interpret the magnitude of the effect. Classically, effects of above 0.2 are considered small, 0.5 medium, and 0.8 large as described by Cohen [34]. While these effect size cutoffs are arbitrary and ideally should be compared to related findings in the literature [35], the lack of similar studies makes this impossible. Using the classical cutoffs, we estimate that from our retrospective data, there were modest strength-to-weight changes with either “Abrahangs Only” or “Max Hangs Only” and large improvements when the protocols were combined in the “Both” group. Since there was no statistical difference between the “Abrahangs Only” and the “Max Hangs Only” groups, and both resulted in a modest effect size on the finger strength: weight ratio, we suggest that the two programs increased finger strength to a similar extent.

Max Hangs training, where 85–95% of 1RM is held for ~ 10s, has long been central to finger strength training. This is consistent with other strength training programs where the increase in strength requires lifting heavy weight [21]. We confirmed the work of others showing that Max Hangs training confers improvements in finger strength compared to climbing alone [2]. The novel submaximal training method we evaluated, “Abrahangs”, was created by professional climber Emil Abrahamsson and his brother Felix based on a protocol from our research group developed using engineered human ligaments [7, 18]. While a positive effect had previously been observed for low-intensity high-frequency loading in in-vitro, this is the first study to examine this training phenomenon in rock climbers. To our surprise, the increase in strength with Abrahangs was equivalent to Max Hangs, and the effects of performing both protocols concurrently were additive.

Strength is dependent on the cross-sectional area of the muscle, the neural activation of the muscle, and the ability to transfer the force from the muscle to the bone to allow movement [22]. Classically, the first two components are the focus of strength training programs. The Phillips group has elegantly dissected these parameters to show that low load training to failure can increase muscle mass without a proportional effect on strength, whereas low volume training at a high load provides a stimulus to increase strength (likely increased neural drive) without a proportional increase in muscle mass [23]. In climbers, muscle mass and neural activation are the major targets of Max Hangs training: build motor programs to recruit the most muscle fibers and provide an anabolic stimulus to the muscles of the forearm. However, because climbing has a much greater reliance on long tendons and the pulleys that align and protect them, it is possible that training designed to better target force transfer within the tendons, fascia, and pulleys may provide a larger stimulus than what would be expected when training muscles of the arms, legs, and trunk.

The “Specificity Principle” states that training adaptations are strongly coupled to the mode, frequency, and duration of the specific exercises performed. Therefore, most performance athletes have not classically used extensive isometric training since all sports are dynamic. Despite this limitation, there have been a all number of studies assessing how isometric training affects performance. Lum et al. demonstrated that training the legs isometrically using sustained contractions (> 1 s) was superior for training maximum strength than plyometric training, but that isometric training using rapid non-sustained contractions was better for targeting rapid force development [24]. The same group showed that both plyometric (dynamic) and isometric resistance training led to improved rate of force development, but that only isometric exercises were effective at improving maximum strength in athletes [25]. These findings are consistent with the current work. The strength gains from hangboarding using low-load, prolonged, isometric contractions were equivalent to the improvements from the shorter, high-load Max Hangs. These data raise the possibility that low-intensity long duration holds may be beneficial in other performance settings.

The 10-minute “Abrahangs” training protocol was designed to allow climbers to stimulate tendon adaptation and promote tendon health and strength through low intensity and frequent loading. The major benefit of the Abrahangs protocol was proposed to be that the training was gentle enough to not interfere with normal climbing and training schedules, while providing the molecular signals to cells within the tendon. This would allow climbers to easily incorporate training into their existing training routine. Thus, climbers do not have to sacrifice training volume or intensity.

The climbers who performed both training protocols together experienced additive strength effects that exceeded the strength gains from either protocol individually. This study validates the role of low intensity training for climbers, particularly when performed concurrently with maximal finger strength training exercises for additive finger strength gains. We postulate that the combination of both protocols would allow athletes to improve in all three components of strength: muscle cross-sectional area and neural drive (Max Hangs), and force transfer (Abrahangs). The results of this retrospective study indicate that Abrahangs are effective for improving finger strength, but future studies are needed to determine the specific adaptations that lead to these improvements, and whether they are protective against injury.

### Limitations

There are several significant limitations to the current study. Importantly, the data were collected retrospectively and therefore the participants were not randomized. As a result, it is possible that they performed their training with significant belief effects that influenced the outcome. The study also relied on self-reported data from climbers who use the Crimpd app. This prevents us from controlling the population demographics and accuracy of the data that were logged. Within this data set, there was also a large variation in the training period (4–16 weeks) as well as in the density of workouts performed by users. As can be seen in Table 2, the large variation in training period was necessary to increase the number of controls (Climbing Only participants). Climbers who are not training their grip frequently also do not test grip strength frequently. The training groups showed similar numbers of climbers in each of the training windows and the demographics (age and climbing ability) were similar between all groups (Table 1). Another issue with self-selected training is that it is completely unclear why the participants picked a specific training program. It is possible that those choosing the Abrahangs protocol had more injuries and started the program with more health centric goals, resulting in selection bias. Further, since the amount of weight transferred from the feet to the fingers in these participants was self-regulated, unsupervised, and not measured, we do not know the load used by those in the Abrahangs group. It is therefore possible that climbers who showed a benefit from the Abrahangs protocol applied greater loads than those who did not benefit. Additionally, the training objectives, motivation, and additional activities or confounding variables were not controlled in this study. Since prior training history and experience were not controlled, we do not know whether these users had prior experience hangboarding or were new users. We also do not know if there were any confounding factors influencing maximum strength when users completed the finger strength assessments (e.g., injury, how well rested the individual was on each test day, mental factors influencing performance). These uncontrolled factors are typical of this type of large retrospective study and should not detract from the conclusions.

The low number of females tested, as indicated in Table 1, was another limitation. We chose to include the data in our analysis because they provide preliminary results on the effectiveness of this protocol in female climbers. Males and females were combined in the analysis, but there were far fewer females on the app who met the inclusion criteria. While climbing is now essentially equally popular in males and females, the subsection of climbers who train finger strength skews heavily male. The literature suggests that women have different general preferences for participating in certain types of exercise and different motivations for performing strength training than men; however, both sexes improve their strength after training, and women may even experience greater relative improvements [36]. The limited data we have on women suggests that Abrahangs are effective in this population; however, much more work needs to be done to recruit more women into future studies.

### Future Studies

To attempt to address the abovementioned limitations and to directly measure the physiological adaptations that may underlie the strength improvements seen in the current study, a prospective study is required to determine: (1) whether this low intensity training can reduce injury risk and improve finger health; (2) whether there are differences in adaptation between men and women; (3) whether randomization (especially having people with belief effects counter to the selected program) affects the outcome; (4) whether standardizing the training program duration affects the adaptations seen in this retrospective report; and (5) whether dynamic imaging can be used to determine whether having the feet on the ground changes the load in the finger flexors and associated pulleys. Any prospective studies should standardize training volume, intensity (including measurement of % body weight used by participants during the Abrahangs), and duration. Such a study should also include both in-person and in-app testing and training to evaluate the protocol in both controlled and real-world training settings. It would also be useful to incorporate direct measurements of finger tendon and pulley cross-sectional area as well since increasing tendon area would decrease the stress of climbing on the finger flexor system, enhance climbing strength and performance, and minimize injury rates.

## Conclusions

A new training technique developed around the ability of tendon cells to adapt, consisting of 10-minute low-intensity long duration holds, has been developed to improve finger strength in rock climbers. This low-intensity long duration hold protocol improved maximum finger strength equivalently to the maximal load training. Combining the two types of training had an additive effect on grip strength. This suggests an important role of force transfer in human strength training and that adding training focused on the tendon to existing strength training programs focused on muscle and neural activation could improve performance.

## Data Availability

All raw data are available on request.

## Abbreviations

IRCRA: International Rock-Climbing Research Association
Abrahangs: a 10-minute low intensity hangboard finger strengthening protocol
Reps: Repetitions
1RM: Maximal weight lifted/held for 1 repetition
